# Promoting physical distancing and not social distancing: When the words matter

**DOI:** 10.1192/j.eurpsy.2021.153

**Published:** 2021-08-13

**Authors:** D. Wasserman

**Affiliations:** National Centre For Suicide Research And Prevention Of Mental Lll-health, Karolinska Institutet, Stockholm, Sweden

**Keywords:** EPA Ethics Committee, Vulnerable groups, physical distancing, Socially connected

## Abstract

As COVID-19 began to plague the world, the term ‘social distancing’ was frequently used, encouraging people to keep a safe physical distance from others to reduce the transmission of the virus. Despite being well-intended, the term has negative implications, further isolating vulnerable groups in society by evoking feelings of rejection and exclusion. For this reason, the members of the European Psychiatric Association Ethics Committee wrote an e-letter in response to an opinion piece published by Science (https://science.sciencemag.org/content/367/6484/1282/tab-e-letters), and also wrote to the European Psychiatry (https://doi.org/10.1192/j.eurpsy.2020.60) and the World Health Organisation explaining why the term ‘physical distancing’ should instead be used among policymakers, governments and the general public. Words are important and carry great meaning. Therefore, by using the term physical distancing and not social distancing the message becomes clear, individuals should remain physically distant but socially connected to protect the vulnerable communities in societies. The World Health Organisation, as well as the Lancet journals, adopting the term physical distancing in replacement of social distancing, was a rewarding and important step in the right direction.

**Disclosure:**

No significant relationships.

